# Smoking Behaviour, Involuntary Smoking, Attitudes towards Smoke-Free Legislations, and Tobacco Control Activities in the European Union

**DOI:** 10.1371/journal.pone.0013881

**Published:** 2010-11-08

**Authors:** Jose M. Martínez-Sánchez, Esteve Fernández, Marcela Fu, Silvano Gallus, Cristina Martínez, Xisca Sureda, Carlo La Vecchia, Luke Clancy

**Affiliations:** 1 Tobacco Control Unit, Institut Català d'Oncologia-ICO, L'Hospitalet de Llobregat, Barcelona, Spain; 2 Department of Clinical Sciences, School of Medicine, Campus of Bellvitge, Universitat de Barcelona, L'Hospitalet de Llobregat, Barcelona, Spain; 3 Cancer Prevention and Control Group, Institut d'Investigació Biomèdica de Bellvitge-IDIBELL, L'Hospitalet de Llobregat, Barcelona, Spain; 4 Department of Epidemiology, Istituto di Ricerche Farmacologiche “Mario Negri”, Milan, Italy; 5 Department of Occupational Medicine, School of Medicine, University of Milan, Milan, Italy; 6 Tobacco Free Research Institute, Dublin, Ireland; Yale University School of Medicine, United States of America

## Abstract

**Background:**

The six most important cost-effective policies on tobacco control can be measured by the Tobacco Control Scale (TCS). The objective of our study was to describe the correlation between the TCS and smoking prevalence, self-reported exposure to secondhand smoke (SHS) and attitudes towards smoking restrictions in the 27 countries of the European Union (EU27).

**Methods/Principal Findings:**

Ecologic study in the EU27. We used data from the TCS in 2007 and from the Eurobarometer on Tobacco Survey in 2008. We analysed the relations between the TCS and prevalence of smoking, self-reported exposure to SHS (home and work), and attitudes towards smoking bans by means of scatter plots and Spearman rank-correlation coefficients (r_sp_). Among the EU27, smoking prevalence varied from 22.6% in Slovenia to 42.1% in Greece. Austria was the country with the lowest TCS score (35) and the UK had the highest one (93). The correlation between smoking prevalence and TCS score was negative (r_sp_ = −0.42, p = 0.03) and the correlation between TCS score and support to smoking bans in all workplaces was positive (r_sp_ = 0.47, p = 0.01 in restaurants; r_sp_ = 0.5, p = 0.008 in bars, pubs, and clubs; and r_sp_ = 0.31, p = 0.12 in other indoor workplaces). The correlation between TCS score and self-reported exposure to SHS was negative, but statistically non-significant.

**Conclusions/Significance:**

Countries with a higher score in the TCS have higher support towards smoking bans in all workplaces (including restaurants, bars, pubs and clubs, and other indoor workplaces). TCS scores were strongly, but not statistically, associated with a lower prevalence of smokers and a lower self-reported exposure to SHS.

## Introduction

The effects of tobacco on health of smokers [1] and non-smokers [2] are well-known and tobacco continues to be the leading preventable cause of death worldwide [3]. Comprehensive smoke-free policies have an important impact on respiratory and cardiovascular diseases [4], [5]. All European Union (EU) countries with the exception of the Czech Republic have ratified the WHO Framework Convention on Tobacco Control [6], and most have implemented tobacco control policies consistent with it [7].

The impact on the health and the anti-smoking climate are important keys in the policy decision for the implementation of smoking bans. Further, the scope of smoking bans which are finally enacted can be influenced by the public opinion and the pressure of specific groups with commercial interests (such as the tobacco industry or the hospitality sector) [8], [9]. In this sense, it is important to provide results about both the effectiveness and the public support of smoke-free policies.

The implementation of comprehensive smoke-free policies decreases the exposure to secondhand smoke (SHS) and their associated health hazards in non-smokers, and may also increase the likelihood of quitting and reducing cigarette consumption among smokers [4], [10]–[15]. Moreover, the support both by the general population and specific groups (ie, hospitality workers) to smoking bans in workplaces increases after their implementation [12], [16]–[18].

The most important policies for tobacco control are price increase, bans or restrictions on smoking in public places, consumer information, bans on tobacco advertisement and promotion of tobacco products, health warnings on boxes of tobacco, and access to treatment for quitting smoking [19]. According to these policies, Joossens and Raw developed in 2006 a Tobacco Control Scale (TCS) in order to quantify the grade and effort of implementation of tobacco control policies in European countries [20].

The objective of this study is to evaluate the correlation between the implementation of tobacco control policies as measured by TCS and smoking prevalence, self-reported exposure to SHS, and attitudes towards smoking bans in the 27 countries of the European Union (EU27).

## Methods

This is an ecologic study with data obtained from different sources, with the country as the unit of analysis. We used data on tobacco control activities in European countries in 2007 as compiled in the TCS [21]. The TCS provides a score for each country based on the level of implementation of smoke-free policies according to the six most important cost-effective policies [19]. We obtained information on smoking prevalence, self-reported exposure to SHS, and attitudes towards smoking bans from the Flash Eurobarometer on Tobacco survey (Flash N° 253) [22]. The Eurobarometer is a cross-sectional study of a representative sample of the adult population (≥15 years old) conducted by the Gallup Organisation in Hungary for the European Commission (commissioned by the Directorate General of Health and Consumers) in the 27 countries of the EU. The fieldwork was conducted in December 2008. In each country, interviews were predominantly carried out via fixed-line telephone. The sample was weighted for socio-demographic variables. The final sample (n = 25,580) was representative of the population aged 15 years and above in each country (about 1,000 persons in each country except Cyprus, Lithuania, and Malta, with approximate 500 respondents)[22].

### Variables

#### Tobacco consumption

Smoking status was obtained using the question from the Eurobarometer: “Regarding smoking cigarettes, cigars or a pipe, which of the following applies to you?”. The possible answers were: “you smoke every day”; “you smoke occasionally”; “you used to smoke but you have stopped”; and “you have never smoked”. We considered two categories of smokers: daily smokers and smokers (daily and occasional smokers).

#### Self-reported exposure to secondhand smoke

Self-reported exposure to SHS at home among non-smokers (former and never smokers) was obtained using the question from the Eurobarometer: “Does any person living with you smoke inside your home?” The possible answers were: “you live alone”; “no one living with you smokes inside the house”; and “someone living with you smokes inside the house”. We considered as exposed to SHS at home individuals who reported to live with a smoker who smokes inside the house. Self-reported exposure to SHS at workplace among smokers and non-smokers was obtained using the question: “At your workplace, how many hours are you exposed to tobacco smoke, on a daily basis?” The possible answers were: “more than 5 hours a day”; “1–5 hour(s)”; “less than 1 hour”; “hardly ever”; and “never exposed”. We considered as exposed to SHS at the workplace individuals who declared to be exposed more than 5 hours a day, 1–5 hour(s), less than 1 hour, and hardly ever. Some analyses were restricted to those reporting to be exposed more than 5 hours a day.

#### Self-reported attitudes towards smoking bans

Information on support to smoke-free policies was obtained using three questions: “Are you in favour of smoking bans in the following places?” 1) restaurants; 2) bars, pubs and clubs; and 3) offices and other indoor workplaces”. The possible answers for these three questions were: “totally opposed”, “somewhat opposed”, “somewhat in favour”, and “totally in favour”. We considered the support to smoke-free policies in the different venues as positive when individuals answered “somewhat in favour” or “totally in favour”.

#### Tobacco control policies

We used data from the Tobacco Control Scale (TCS) of 2007 [21]. The TCS was developed by an experts' working group from the European Network for Smoking Prevention (ENSP). The scale was developed by means of a questionnaire that was sent to the ENSP correspondents within the countries. The score of each policy was weighted by its reported effectiveness, based on existing research and the discussion of a panel of experts on tobacco control. The six policies and their corresponding score are: price increases through higher taxes on tobacco products (maximum 30 points); bans/restrictions on smoking in public and work places (maximum 22 points); better consumer information including public information campaigns, media coverage, and publicising of research findings (maximum 15 points); comprehensive bans on the advertising and promotion of all tobacco products, logos and brand names (maximum 13 points); large direct health warning labels on cigarettes' boxes and other products (maximum 10 points); and treatment to help dependent smokers quit, including increased access to medications (maximum 10 points). The maximum score of the TCS is 100 points, indicating a full implementation of all the strategies considered. Other data (the price of 20 cigarettes, the tobacco legislation database, etc.) were obtained from other sources, and were used to score the scale (see references 20 and 21 for more details).

### Statistical analysis

We analysed the association between the TCS score and smoking prevalence, self-reported exposure to SHS, and attitudes towards smoking bans by means of scatter-plots and Spearman rank-correlation coefficients (r_sp_). To adjust for multiple comparisons testing, we fixed the α error to 1%. We hence calculated the 99% confidence intervals (99% CI) of the Spearman coefficients. We also analysed the relation between the score of each six policies and prevalence.

### Ethics statement

This investigation was based in secondary data from available databases and did not involved humans. Hence, no approval of the Bellvitge University Hospital research and ethics committee was necessary.

## Results

The prevalence of smokers was 31.5% (95% CI: 30.9%, 32.1%) in EU27, varying from 22.6% in Slovenia to 42.1% in Greece. The prevalence of never smokers was 46.3% (95% CI: 45.7%, 46.9%) in EU27, varying from 39.1% in Denmark to 58.0% in Cyprus. The prevalence of self-reported exposure to SHS at home among non-smokers was 13.6% (95% CI: 13.1%, 14.1%) in EU27, varying from 2% in Finland to 31.4% in Cyprus. The prevalence of self-reported exposure to SHS at work was 21.2% (95% CI: 20.7%, 21.7%) in EU27, varying from 11.8% in Italy to 41.8% in Cyprus.

The percentage of individuals who supported the implementation of smoke-free policies in restaurants varied from 62.4% in Austria to 95.0% in Italy; in bars, pubs, and clubs, it varied from 43.7% in Netherlands to 93.1% in Italy; and in offices and other indoor workplaces varied from 66.2% in Cyprus to 94.7% in Italy.

Austria was the country with the lowest score in TCS (35) and the UK had the highest one (93). The countries that have higher scores in TCS (UK, Ireland, Malta, and Sweden; scores >60) showed relatively low smoking prevalence (less than 28.8%) and low prevalence of self-reported exposure to SHS (less than 13.8% at home and 23.4% at work). In the countries with lower scores in the TCS (Czech Rep., Germany, Luxemburg, Greece, and Austria; scores ≤40) the smoking prevalence was relatively high (over 30%), as well as the prevalence of self-reported exposure to SHS (between 15 and 30% at home; and between 15 and 36% at work).

**Figure 1:**
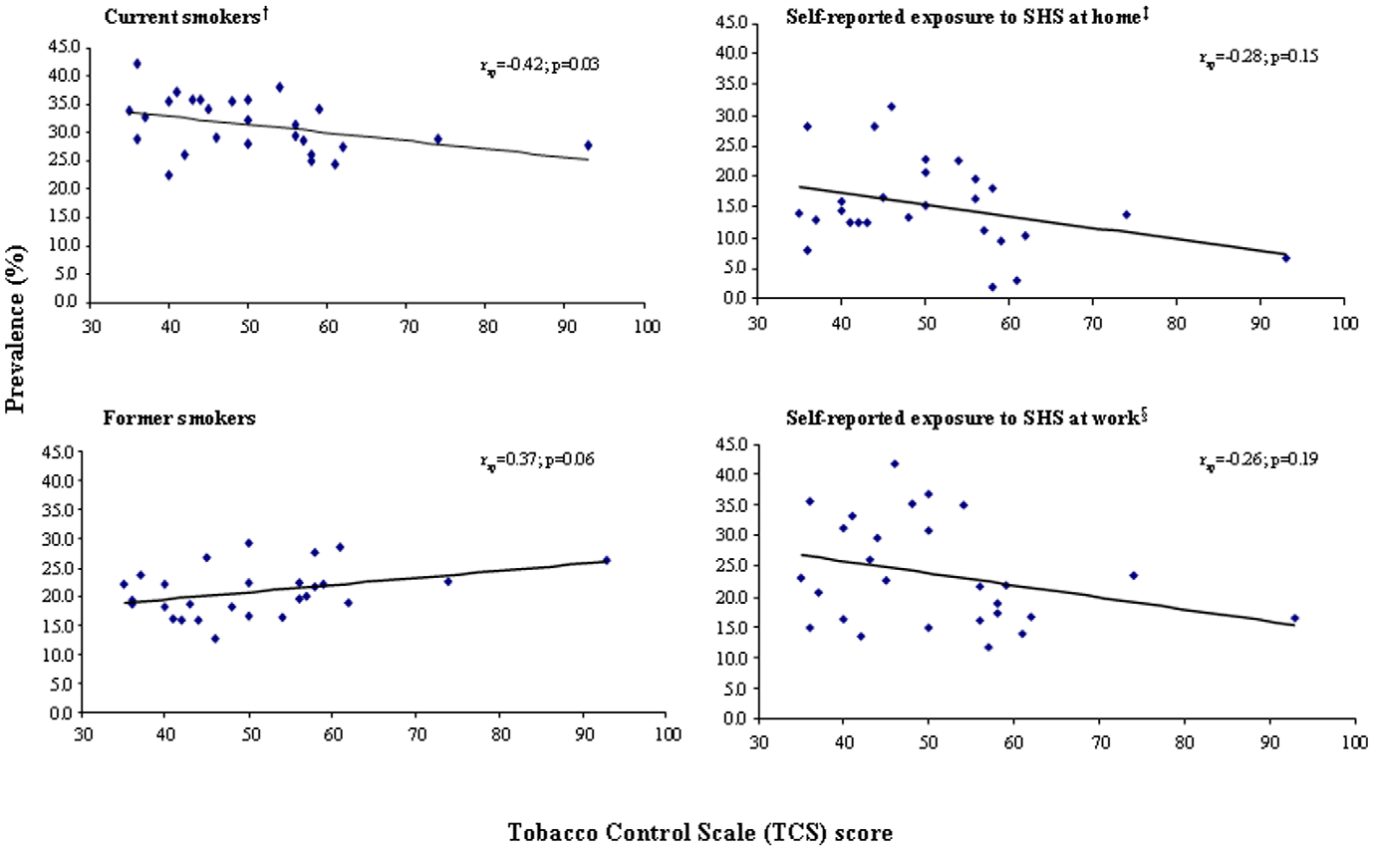
Correlation between Tobacco Control Scale score and prevalence of smoking status (current smokers and former smokers) and self-reported exposure to secondhand smoke (SHS) at home and at work in the European Union (EU27). r_sp_: Spearman's rank correlation coefficient. † Current smokers: daily and occasionally smokers. ‡ Only non-smokers' exposure to SHS at home. § Smokers and non-smokers' exposure to SHS at work.

There was an inverse association between TCS score and the prevalence of occasional and daily smokers (r_sp_ = −0.42, 99% CI: −0.75, 0.08; p = 0.03) and a direct association with the prevalence of former smokers (r_sp_ = 0.37, 99% CI: −0.14, 0.72; p = 0.06) (figure 1). Self-reported exposure to SHS at home and work was inversely associated with TCS score, but statistically non-significant (table 1). There was an inverse association of borderline statistical significance between TCS score and self-reported exposure to SHS at work more than 5 hours (r_sp_ = −0.43, 99% CI: −0.76, 0.07, p = 0.02). The correlation coefficients were similar after excluding those countries showing extreme values (data not shown). Furthermore, since the prevalence of smokers and the proportion of exposed to SHS were highly correlated (r_sp_ = 0.46 for SHS exposure at home and r_sp_ = 0.63 for SHS exposure > 5h at work) we considered the correlation between self-reported SHS exposure and TCS scale in separate strata of prevalence of smokers. The correlation coefficients remained moderately high (though statistically non-significant) in the strata of countries with prevalence of smokers <30% (r_sp_ = −0.35 for SHS exposure at home and r_sp_ = −0.25 for SHS exposure >5 h at work) whereas in the strata of prevalence of smokers ≥30% the correlation coefficients were close to 0.

**Table 1:** Correlation (r_sp_) and 99% confidence intervals (CI) between Tobacco Control Scale (TCS) score, its six components, and prevalence of smoking (current smokers and former smokers), self-reported exposure to secondhand smoke (SHS) at home and at work, and attitudes towards smoking bans.

	TCS	Price	Public place bans	Public information campaign spending	Advertising bans	Health warnings	Treatment
**Smoking status**							
Smokers (daily and occasionally)	−0.42 (−0.75, 0.08)	−0.22 (−0.63, 0.3)	−0.31 (−0.69, 0.2)	−0.28 (−0.67, 0.24)	−0.5 (−0.79, −0.02)	−0.42 (−0.75, 0.07)	−0.07 (−0.53, 0.43)
*p-value*	0.03	0.28	0.11	0.20	0.009	0.03	0.72
Smokers (daily)	−0.32 (−0.69, 0.19)	−0.22 (−0.63, 0.3)	−0.27 (−0.66, 0.25)	−0.1 (−0.56, 0.4)	−0.42 (−0.75, 0.08)	−0.45 (−0.76, 0.05)	0.04 (−0.45, 0.51)
*p-value*	0.11	0.28	0.18	0.65	0.03	0.02	0.84
Former smokers	0.37 (−0.14, 0.72)	0.14 (−0.37, 0.58)	0.21 (−0.3, 0.63)	0.3 (−0.21, 0.68)	0.32 (−0.19, 0.7)	0.09 (−0.41, 0.55)	0.54 (0.07, 0.81)
*p-value*	0.06	0.49	0.29	0.17	0.10	0.65	0.004
**Exposure to SHS**							
at home‡	−0.28 (−0.67, 0.23)	−0.26 (−0.66, 0.25)	−0.27 (−0.67, 0.24)	−0.17 (−0.6, 0.34)	0.03 (−0.46, 0.51)	−0.31 (−0.69, 0.2)	−0.17 (−0.6, 0.34)
*p-value*	0.15	0.19	0.17	0.45	0.88	0.12	0.40
at work	−0.26 (−0.66, 0.25)	−0.05 (−0.52, 0.45)	−0.31 (−0.69, 0.2)	−0.31 (−0.69, 0.2)	−0.14 (−0.58, 0.37)	−0.28 (−0.67, 0.23)	0.01 (−0.48, 0.49)
*p-value*	0.19	0.82	0.12	0.15	0.49	0.15	0.98
at work (more than 5 hours)	−0.43 (−0.76, 0.07)	−0.12 (−0.57, 0.39)	−0.43 (−0.76, 0.06)	−0.32 (−0.69, 0.19)	−0.23 (−0.64, 0.29)	−0.28 (−0.67, 0.23)	−0.33 (−0.7, 0.18)
*p-value*	0.02	0.56	0.02	0.14	0.26	0.16	0.08
**Attitudes to smoking bans** *							
smoking bans in restaurants	0.47 (−0.02, 0.78)	0.26 (−0.25, 0.66)	0.61 (0.18, 0.84)	0.25 (−0.26, 0.65)	0.15 (−0.36, 0.59)	0.38 (−0.13, 0.73)	0.08 (−0.42, 0.54)
*p-value*	0.01	0.18	0.001	0.25	0.46	0.05	0.70
smoking bans in bars, pubs and clubs	0.5 (0.02, 0.79)	0.18 (−0.33, 0.61)	0.66 (0.26, 0.87)	0.31 (−0.2, 0.69)	0.31 (−0.2, 0.69)	−0.04 (−0.51, 0.45)	0.25 (−0.26, 0.65)
*p-value*	0.008	0.37	<0.001	0.15	0.11	0.85	0.21
smoking restrictions in offices and other indoor workplaces	0.39 (−0.11, 0.73)	0.06 (−0.43, 0.53)	0.46 (−0.03, 0.77)	0.14 (−0.37, 0.58)	0.11 (−0.39, 0.56)	0.19 (−0.32, 0.62)	0.05 (−0.44, 0.52)
*p-value*	0.12	0.75	0.02	0.52	0.60	0.35	0.81

Price: price increases through higher taxes on tobacco products (maximum 30 points); Public place bans: bans/restrictions on smoking in public and work places (maximum 22 points); Public information campaign spending: better consumer information including public information campaigns, media coverage, and publicising of research findings (maximum 15 points); Advertising bans: comprehensive bans on the advertising and promotion of all tobacco products, logos and brand names (maximum 13 points); Health warnings: large direct health warning labels on cigarettes' boxes and other products (maximum 10 points); Treatment: treatment to help dependent smokers quitting, including increased access to medications (maximum 10 points).

r_sp_: Spearman's rank correlation coefficient.

*Attitudes to smoking bans (somewhat in favour or totally in favour).

‡ Only non-smokers' exposure to SHS at home.

**Figure 2:**
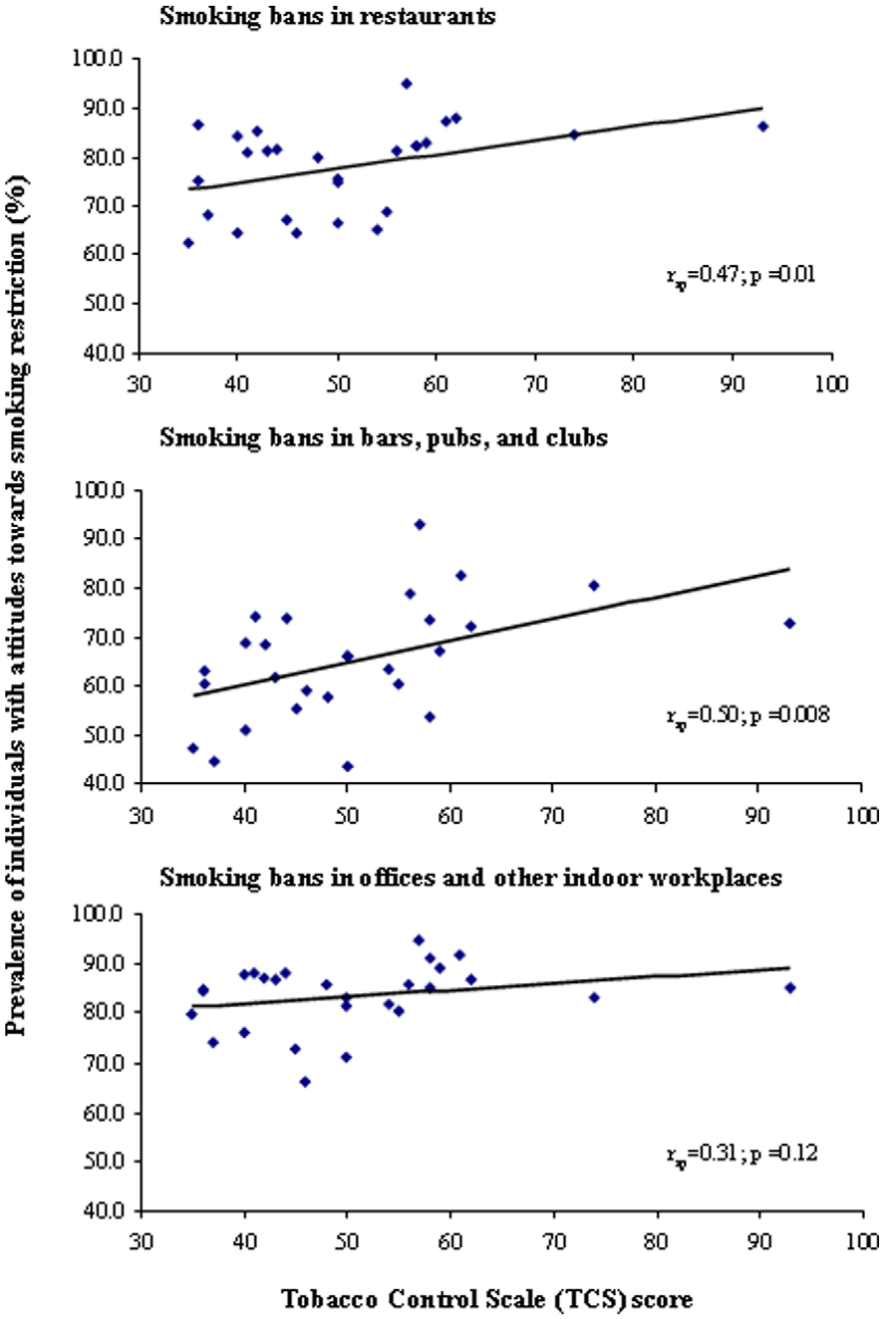
Correlation between Tobacco Control Scale (TCS) and attitudes to smoking bans (somewhat in favour or totally in favour) in the European Union (EU27). r_sp_: Spearman's rank correlation coefficient.

When we excluded the policy on bans/restrictions in public and workplaces from the TCS score, the associations remained inverse and statistically non significant (exposure at home r_sp_ = −0.24, 99% CI: −0.65, 0.27; p = 0.24 and exposure at work r_sp_ = −0.16, 99% CI: −0.60, 0.35; p = 0.43). Those countries with high scores in TCS showed higher percentage of favourable attitudes towards smoking bans in all workplaces (restaurants, bars, pubs, clubs, and other indoor workplaces) (figure 2). The percentage of favourable attitudes towards smoking bans was higher in countries with lower smoking prevalence (in restaurants, r_sp_ = −0.56, 99% CI: −0.82, −0.11; p = 0.002, in bars, pubs, and clubes, r_sp_ = −0.24, 99% CI: −0.65, 0.27; p = 0.22, and in other indoor workplaces, r_sp_ = −0.25, 99% CI: −0.65, 0.26; p = 0.20). Additionally, we analysed the correlation between each of the six specific policies and smoking prevalence, self-reported exposure to SHS, and attitudes towards smoking bans (table 1). Implementation of advertising bans was inversely correlated with active smoking (r_sp_ = −0.50, 99% CI: −0.79, −0.02; p = 0.009), and high implementation of treatments for quitting smoking was directly correlated with prevalence of former smokers (r_sp_ = 0.54, 99% CI: 0.07, 0.81; p = 0.004). Finally, smoking bans in public places were directly correlated with support to smoking bans in restaurants (r_sp_ = 0.61, 99% CI: 0.18, 0.84; p = 0.001), in bars, pubs, and clubs (r_sp_ = 0.66, 99% CI: 0.26, 0.87; p<0.001), and in offices and other indoor workplaces (r_sp_ = 0.46, 99% CI: −0.03, 0.77; p = 0.02).

## Discussion

European countries with more developed tobacco control policies as indicated by higher TCS scores (price increase, bans or restrictions on smoking in public places, consumer information, bans on tobacco advertisement and promotion of tobacco products, health warning on tobacco boxes, and access to treatment for quitting smoking) were strongly, but not statistically, associated with a lower population prevalence of smokers and a lower self-reported exposure to SHS at home and work. Moreover, there is widespread support to smoking restrictions in all public places in these countries, and tobacco control policies are more advanced.

A study in 18 European countries found a positive association between the quit rate and the TCS score; this relation was similar in high and low educational levels [23]. In our study, more advertising bans were inversely correlated (in the limit of significance), with smoking prevalence and the availability of treatments for quitting was directly correlated with the prevalence of former smokers. One study in adolescents from 29 European countries suggested that specific policies on price, public bans, and advertising bans may help to prevent starting smoking and to decrease smoking prevalence in young boys [24]. However, another study that assessed the relationship between TCS score and motivation (stages of change) of the smokers to quit in five European countries found no association [25].

Our study shows an inverse but not statistically significant correlation between the TCS score and the self-reported exposure to SHS at home and at work. The correlations were high between the score of smoking bans on public places and self-reported exposure to SHS at work and between health warnings policies and self-reported exposure to SHS at home, although these correlations did not achieved statistical significance at 1% level. However, the intensity of exposure at work was highly correlated with smoking ban in public places in the limit of the significance. Longitudinal studies have found a decrease in SHS at work after the implementation of comprehensive smoke-free laws in the general population and in hospitality workers [4].

We found a direct correlation between the TCS score and the support towards smoking bans restrictions in all workplaces, including restaurants, bars, pubs, and clubs. The correlation between the TCS score and support towards smoking bans was mainly due to the correlation with public place bans. Price increases, public information campaign spending, advertising bans, and health warnings showed moderate correlations. Longitudinal studies from different European countries (Ireland [16], Scotland [17], and Spain [18]) have reported an increase on the support to smoking bans after the implementation of national smoke-free laws in all workplaces including restaurants, bars, pubs, and clubs by the general population and also by hospitality workers [12], [16]–[18]. This could be partially explained because these countries have banned tobacco advertising and launched more media campaigns (TV, radio, newspapers, etc.) about the adverse effects of exposure to SHS on health of non-smokers [18]. Finally, we found a direct association between the treatment component of the TCS and the prevalence of former smokers. Although the weight of treatment in the total TCS score is limited (10 out of 100 points), the impact on quitting seems to be important at the ecological level. Further, there is still debate about the quantifiably impact of pharmacological treatments to control the tobacco epidemic [26], [27].

### Strengths and limitations of the study

This was an ecological study, and consequently, any causal relationship between tobacco control policies and the outcomes assessed (smoking prevalence, self-reported SHS exposure, and attitudes towards smoking bans) is difficult to establish. Indeed, more strict smoking control policies may reflect, rather than cause, more advanced attitudes towards tobacco smoking and tobacco control on a population level. However, the results of our study are in agreement with other studies showing a reduction in smoking prevalence and SHS exposure, and an increase in the support of national bans after smoke-free policies [4]. We are not trying to infer the relationship at the individual level but simply assessing an ecological effect [28].

Another limitation of our study is the lack of information about the stage of the tobacco epidemic across the different countries [29]. This information could help to better understand the relationships studied. Lopez et al. [29] already suggested that smoke-free public places and transports are common achievements at stage III of the epidemic but not smoke-free workplaces that are implemented later at stage IV. The use of self-reported data from questionnaires could be a source of bias, although self-reports on smoking status have acceptable validity [30]. On the other hand, the delay between the TCS (from 2007) and the Eurobarometer survey (from the end of 2008) provides an adequate time-frame (less than two years) to observe the potential effects of tobacco control policies on smoking behaviour and self-reported exposure to SHS. Finally, the small sample size in each country and the lack of information about the number of cigarettes smoked per day and number of hours exposed to SHS at home could be another limitation. However, the sample design of the Eurobarometer guarantees the representativeness by country [22].

In conclusion, this study shows at the ecological level that countries with higher score in the TCS have higher support towards smoking bans in workplaces.

## References

[1] US Department of Health and Human Services (2004). The health consequences of smoking..

[2] US Department of Health and Human Services (2006). The health consequences of involuntary exposure to tobacco smoke: a report of the Surgeon General..

[3] World Health Organization (WHO) (2008). MPOWER. WHO Report on the Global tobacco epidemic..

[4] IARC Working Group (2009). IARC handbooks of cancer prevention: tobacco control. Vol. 13. Evaluation of the effectiveness of smoke-free polices..

[5] Callinan JE, Clarke A, Doherty K, Kelleher C (2010). Legislative smoking bans for reducing secondhand smoke exposure, smoking prevalence and tobacco consumption.. Cochrane Database of Systematic Reviews 2010, Issue 4. Art. No.: CD005992..

[6] http://www.who.int/fctc/signatories_parties/en/index.html.

[7] Jaakkola MS, Jaakkola JJ (2006). Impact of smoke-free workplace legislation on exposures and health: possibilities for prevention.. Eur Respir J.

[8] Muggli ME, Lockhart NJ, Ebbert JO, Jiménez-Ruiz CA, Riesco-Miranda JA (2010). Legislating tolerance: Spain's national public smoking law.. Tob Control.

[9] Sebrie EM, Glantz SA (2007). “Accommodating” smoke-free policies: tobacco industry's Courtesy of Choice programme in Latin America.. Tob Control.

[10] Chapman S, Borland R, Scollo M, Brownson RC, Dominello A (1999). The impact of smoke-free workplaces on declining cigarette consumption in Australia and the United States.. Am J Public Health.

[11] Anonymous (2005). Ireland's smoking ban is an admirable achievement.. Lancet.

[12] Fong GT, Hyland A, Borland R, Hammond D, Hastings G (2006). Reductions in tobacco smoke pollution and increases in support for smoke-free public places following the implementation of comprehensive smoke-free workplace legislation in the Republic of Ireland: findings from the ITC Ireland/UK Survey.. Tob Control.

[13] Gallus S, Zuccaro P, Colombo P, Apolone G, Pacifici R (2007). Smoking in Italy 2005-2006: effects of a comprehensive National Tobacco Regulation.. Prev Med.

[14] Fichtenberg CM, Glantz SA (2002). Effect of smoke-free workplaces on smoking behaviour: systematic review.. BMJ.

[15] Goodman PG, Haw S, Kabir Z, Clancy L (2009). Are there health benefits associated with comprehensive smoke-free laws.. Int J Public Health.

[16] Pursell L, Allwright S, O'Donovan D, Paul G, Kelly A (2007). Before and after study of bar workers' perceptions of the impact of smoke-free workplace legislation in the Republic of Ireland.. BMC Public Health.

[17] Hilton S, Semple S, Miller BG, MacCalman L, Petticrew M (2007). Expectations and changing attitudes of bar workers before and after the implementation of smoke-free legislation in Scotland.. BMC Public Health.

[18] Martinez-Sanchez JM, Fernandez E, Fu M, Perez-Rios M, Schiaffino A (2010). [Changing expectations and attitudes of hospitality workers after the implementation of the Spanish smoking law].. Gac Sanit.

[19] World Bank Tobacco control at a glance, Washington DC, 2003.. http://www.worldbank.org/tobacco.

[20] Joossens L, Raw M (2006). The Tobacco Control Scale: a new scale to measure country activity.. Tob Control.

[21] Joossens L, Raw M Progress in Tobacco Control in 30 European Countries, 2005 to 2007.. http://www.ensp.org/files/30_european_countries_text_final.pdf.

[22] European Commision The Gallup Organisation. Survey on Tobacco.. http://ec.europa.eu/health/ph_determinants/life_style/Tobacco/keydo_tobacco_en.htm.

[23] Schaap MM, Kunst AE, Leinsalu M, Regidor E, Ekholm O (2008). Effect of nationwide tobacco control policies on smoking cessation in high and low educated groups in 18 European countries.. Tob Control.

[24] Hublet A, Schmid H, Clays E, Godeau E, Gabhainn SN (2009). Association between tobacco control policies and smoking behaviour among adolescents in 29 European countries.. Addiction.

[25] Thyrian JR, Panagiotakos DB, Polychronopoulos E, West R, Zatonski W (2008). The relationship between smokers' motivation to quit and intensity of tobacco control at the population level: a comparison of five European countries.. BMC Public Health.

[26] Chapman S, Mackenzie R (2010). The global research neglect of unassisted smoking cessation: causes and consequences.. PLoS Med.

[27] West R, McNeill A, Britton J, Bauld L, Raw M (2010). Should smokers be offered assistance with stopping?. Addiction.

[28] Diez-Roux AV (2000). Multilevel analysis in public health research.. Annu Rev Public Health.

[29] Lopez AD, Collishaw NE, Piha T (1994). A descriptive model of the cigarette epidemic in developed countries.. Tob Control.

[30] Gorber SC, Schofield-Hurwitz S, Hardt J, Levasseur G, Tremblay M (2009). The accuracy of self-reported smoking: a systematic review of the relationship between self-reported and cotinine-assessed smoking status.. Nicotine Tob Res.

